# Psychosocial Cardiological Schedule-Revised (PCS-R) in a Cardiac Rehabilitation Unit: Reflections Upon Data Collection (2010–2017) and New Challenges

**DOI:** 10.3389/fpsyg.2020.01720

**Published:** 2020-07-14

**Authors:** Nicolò Granata, Ekaterina Nissanova, Valeria Torlaschi, Marina Ferrari, Martina Vigorè, Marinella Sommaruga, Elisabetta Angelino, Claudia Rizza, Alessandra Caprino, Antonia Pierobon

**Affiliations:** ^1^Psychology Unit, Istituto di Montescano, Istituti Clinici Scientifici Maugeri IRCCS, Pavia, Italy; ^2^Department of Cardiac Rehabilitation, Istituto di Montescano, Istituti Clinici Scientifici Maugeri IRCCS, Pavia, Italy; ^3^Psychology Unit, Istituto di Camaldoli, Istituti Clinici Scientifici Maugeri IRCCS, Milan, Italy; ^4^Psychology Unit, Istituto di Torino, Istituti Clinici Scientifici Maugeri IRCCS, Turin, Italy; ^5^Gruppo Multimedica Spa-IRCCS, Milan, Italy

**Keywords:** psychosocial screening, psychological interview, cardiac rehabilitation, nursing, interdisciplinary intervention, rehabilitation medicine

## Abstract

**Introduction:**

The Psychosocial Cardiological Schedule (PCS) was developed as a screening tool for patients undergoing cardiac rehabilitation (CR) to detect clinically relevant psychosocial/cognitive problems requiring psychological assessment/intervention. Filled out by a trained nurse, it classifies patients according to their need or not for a psychological interview and intervention provided by the psychologist (PCS-Yes vs. PCS-No).

**Aims:**

The main aim was to compare PCS data collected, respectively, in 2010 and 2017, regarding patients’ socio-demographic characteristics, clinical variables, and the inclusion criteria for psychological counseling. Subsequently, the original Italian PCS was revised and an English version of the schedule was provided [PCS-Revised (PCS-R)].

**Results:**

28 patients (aged 53.5 + 12.6 years, M = 20) of the 87 recruited in 2010 vs. 35 (aged 64.9 + 12.7 years, M = 28) of the 83 recruited in 2017 met the criteria for PCS-Yes: age < 55 years, social problems (living alone, no social support), manifest psychological/behavioral problems, suspected neuropsychological disorders, low prescription adherence, inadequate disease awareness. Comparing the two samples (2010 vs. 2017), clinical variables were similar, and the need for a psychological interview did not differ substantially (32.2 vs. 42.2%), but age increased significantly (PCS-Yes: 53.5 ± 12.6 vs. 64.9 ± 12.7 years, *p* = 0.001; PCS-No: 68.3 ± 8.0 vs. 75.0 ± 7.7 years, *p* = 0.0001). A significant increase was observed in the recommendation for neuropsychological assessment (3.6 vs. 25.7%, *p* = 0.02) to confirm eventual cognitive deficits. These results, the clinical experience, and the recent evidences from literature led to the PCS-R, incorporating a psychosocial screening, a psychological/neuropsychological deeper assessment, and a recommendation for a specific intervention to be carried out either during rehabilitation or in outpatient services.

**Conclusion:**

The data comparison highlight changes in the cardiac population, which is aging and more frequently requires neuropsychological assessment. The PCS-R could be considered in clinical practice as a useful screening tool to implement a timely coordinated interdisciplinary intervention, comprehensive of specific and tailored psychotherapeutic techniques.

## Introduction

Cardiac rehabilitation (CR) is a comprehensive multidisciplinary program individually tailored to the needs of patients with cardiovascular (CV) disease (Smith et al., 2006; Balady et al., 2007). The objectives in the medium and long term are to reduce the risk of subsequent CV events (morbidity and mortality) and delay the progression of atherosclerosis, of the underlying cardiopathy and clinical deterioration (Piepoli et al., 2016; Fattirolli et al., 2018). The overall focus is on improving daily function and reducing CV risk factors. CR includes interventions to lower blood pressure and improve lipid and diabetes mellitus control, tobacco cessation, behavioral counseling, and graded physical activity (Anderson and Taylor, 2014; Pogosova et al., 2015; Piepoli et al., 2016; Servey and Stephens, 2016; Albus et al., 2019a; Long et al., 2019). Many CR guidelines and position statements recommend screening for psychosocial risk factors, although there is wide variation in the recommended factors and recommended screening tools (Jackson et al., 2017; Sommaruga et al., 2018; Albus et al., 2019b). National and European CV prevention and rehabilitation guidelines (Pogosova et al., 2015; Griffo et al., 2016; Fattirolli et al., 2018) recommend multidisciplinary interventions involving nurses, physiotherapists, dieticians, and psychologists in collaboration with cardiologists. The core tasks to be performed by each single team member have been detailed in recent position papers (Piepoli et al., 2016; Fattirolli et al., 2018).

The increased participation in CR of complex, elderly comorbid patients with psychological and neuropsychological problems raises the need to refine the screening models used to identify patients requiring psychological support (Gellis and Kang-Yi, 2012; Forman and Wenger, 2013; Cameron et al., 2017). This is matched by a growing interest on the part of nurses to improve their own skills in identifying and managing patient psychosocial difficulties, within the time limits of their workload in a CR setting (Turner et al., 2017; Winder et al., 2017). The synergy between these two professional figures—nurse and psychologist—can be seen as a valid reflection of the multidisciplinary nature of CR today. The CR short-term goals are clinical stability, limitation of the physiological and psychological consequences of CV disease, and overall improvement of the patient’s functional capacity and degree of autonomy, independence, and quality of life. In CR, the psychologist’s specific contribution is to help identify and implement, through health and clinical psychology, adequate diagnostic-therapeutic pathways (Pogosova et al., 2015; Richards et al., 2018; Sommaruga et al., 2018; Albus et al., 2019b; Pedersen and Doyle, 2019).

The starting point of an appropriate psychological intervention in CR is to early identify problems or needs that may indicate the necessity for specific psychological support according to the latest cardiac guidelines (Giannuzzi, 2016; Piepoli et al., 2016; Jbilou et al., 2019; McPhillips et al., 2019).

In this process, the dialog between the psychologist and the other healthcare professionals allows a useful and timely collection of information and observations that are registered and codified. To this end, the Psychology Unit in collaboration with the Cardiac Rehabilitation Department of ICS Maugeri – Montescano (PV) developed a Psychosocial Cardiological Schedule (PCS) aimed to detect indicators of psychological, behavioral, social, and cognitive problems/needs. The psychologists, who have been working at the Cardiac Rehabilitation Department for many years, previously trained the nursing staff to detect patients’ psychosocial manifestations/needs, cardiac behavioral risk factors, and problems in dealing with illness management. A trained nurse fills in the PCS, providing screening information and a written clinical report for care pathways management according to the need or not for a psychological interview and intervention (Pierobon et al., 2012). The screening process considers data from nursing and medical records, observational data related to the patient’s behavior in the hospital ward, information about the patient given by the caregiver, and any other clinical data available.

The first version of the PCS (Pierobon et al., 2012) was in the Italian language. The sections required the compilation of demographic, anamnestic, clinical-psychosocial data, and information related to both the knowledge and the management of the disease. It is a screening schedule, structured as a checklist, which indicated the selection criteria for a deeper psychological interview. These criteria, outlined in gray in the PCS, are described below in Section “Procedure.” More precisely, any psychological/psychiatric problems and risk factors detected in the medical history constitute a specific indication for psychological assessment only when they have a clinically relevant impact on the patient’s current health status (Graham et al., 2007; Piepoli et al., 2016). The age criterion (arbitrarily set at ≤ 55 years) responded to the clinical need to evaluate the impact of the disease in a phase of the life cycle that is still fully active from a personal, family, and work status point of view and therefore involves a process of complex psychological adaptation (Olano-Lizarraga et al., 2016; Helgeson and Zajdel, 2017). The other selection criteria were decided based on scientific evidence from the literature (Sommaruga et al., 2003; Lichtman et al., 2008; Albus et al., 2019a).

The main aim of the present study was to compare the PCS data collected in January–February 2010 with those collected in January–February 2017, focusing on the differences between patients’ socio-demographic characteristics, clinical variables, and the inclusion criteria for psychological interview. Subsequently, a second aim was to update the Italian version of the PCS in light of the reflections made upon PCS data comparison, as well as the most recent CR guidelines and position papers. Besides, an English translation of the revised PCS was provided [PCS-Revised (PCS-R)].

## Materials and Methods

### Procedure

The data analysis of this retrospective comparative study was performed considering all cardiac patients consecutively admitted for inpatient rehabilitation to the Cardiac Rehabilitation Department of ICS Maugeri – Montescano (Pavia), Italy in 2 months: January–February 2010 and January–February 2017. All patients underwent a screening evaluation with the original version of PCS (Pierobon et al., 2012) filled in by a trained nurse, within 2 days of admission. Patients were divided into two groups based on whether they met the selection criteria for a deeper psychological interview (PCS-Yes) or not (PCS-No). The selection criteria for PCS-Yes were: age (≤55 years), absence or lack of social and/or family support, current smoking, current manifestation of at least one psychological/psychiatric symptom, inadequate illness awareness, presence of a neuropsychological disorder, and poor adherence to clinical prescriptions. Patients who met one of the aforementioned criteria underwent a planned psychological interview (and/or test evaluation) and the related psychological intervention. All patients were enrolled in the CR program following an acute cardiac event (acute myocardial infarction, myocardial revascularization, cardiac surgery, and acute pulmonary edema) or an instability of a chronic condition, in accordance with the CR guidelines (Piepoli et al., 2016; Fattirolli et al., 2018). Patients signed an informed consent for all procedures and explanations at the admittance. The study was approved by our Institutional Review Board and Central Ethics Committee of the ICS Maugeri SpA SB (CEC) (approval number: CEC N.927, 27/06/2013).

After data collection and analysis, the original Italian version of the PCS (Pierobon et al., 2012) was modified and integrated (Supplementary Data Sheet S1) based on the results of the PCS data comparison, the evidence from the psychosocial literature about CV diseases (Pogosova et al., 2015; Sommaruga et al., 2018; Albus et al., 2019b), the scientific evidence about screening tools in CR (Jackson et al., 2017), and the most recent CR guidelines (Piepoli et al., 2016; Fattirolli et al., 2018). Besides, an English version of the revised PCS was prepared (Supplementary Data Sheet S2).

### Statistical Analysis

Descriptive statistics are reported as mean ± standard deviation (SD) for continuous variables and as number (percentage) for discrete variables. Comparisons between groups (2010–2017) for continuous variables were assessed by Student’s *t*-test for independent samples, while categorical variables were assessed by chi-square test. The analysis focused on differences between patients’ socio-demographic characteristics, clinical variables, and the inclusion criteria for a psychological interview. All statistical tests were two-tailed and statistical significance was set at *p* < 0.05. All analyses were carried out using the SAS/STAT statistical package, release 9.2 (SAS Institute Inc., Cary, NC, United States).

## Results

Table 1 reports the PCS data on socio-demographic and clinical characteristics of the two study samples (2010–2017). We found a significantly lower mean age in 2010 compared to 2017 (PCS-Yes, 53.5 ± 12.6 vs. 64.9 ± 12.7, *p* < 0.001; PCS-No, 68.3 ± 8.0 vs. 75.0 ± 7.7, *p* < 0.0001). Furthermore, the number of male patients increased significantly from 2010 to 2017 (71.4 vs. 80%, χ^2^ = 8.43, *p* = 0.0037). As to patients’ current and past clinical cardiac characteristics, social status, and risk factors, no significant differences were found between the two samples. Table 2 shows the frequency distribution of the selection criteria for in-depth psychological interview (PCS-Yes) in the two samples. 28 patients of the 87 recruited in 2010 and 35 of the 83 recruited in 2017 met the criteria for PCS-Yes. The indication for psychological interviews based on anxiety symptoms was significantly higher in 2010 (32.1%) than in 2017 (11.4%). Instead, the indication for neuropsychological assessment due to cognitive disorders was significantly lower in 2010 (3.6%) than in 2017 (14.3%).

**TABLE 1:** Socio-demographic and clinical results of the two samples.

	2010 (*n* = 87)		2017 (*n* = 83)	
	PCS-YES (*n* = 28)	PCS-NO (*n* = 59)	PCS-YES (*n* = 35)	PCS-NO (*n* = 48)
	M(SD)	M(SD)	M(SD)	M(SD)
Age	53.5 ± 12.6^a^	66.6 ± 8.0^c^	64.9 ± 12.7^b^	75.0 ± 7.7^d^
Duration of cardiac illness (years)	7.7 ± 5.5	9.0 ± 7.9	4.9 ± 7.4	10.0 ± 9.3
BMI	27.4 ± 6.9	26.7 ± 3.7	26.5 ± 5.9	25.7 ± 4.4

	***n* (%)**	***n* (%)**	***n* (%)**	***n* (%)**

Gender (male)	20 (71.4)	24 (40.7)	28 (80.0)	32 (66.7)
Family status (married)	15 (53.6)	48 (81.4)	19 (54.3)	28 (58.3)
Current occupation (yes)	12 (42.8)	4 (6.8)	9 (25.8)	3 (6.3)
Dwelling status (living alone)	9 (32.1)	7(11.9)	5 (14.3)	7 (14.6)
Social problems (yes)	3 (10.7)	1 (1.7)	–	–
Current smoking habit	8 (28.6)	12 (20.3)	9 (25.7)	7 (14.6)
**Anamnesis risk factors**				
Smoking	18 (64.3)	30 (50.8)	16 (45.7)	11 (22.9)
Dyslipidemia	12 (42.9)	35 (59.3)	13 (37.1)	19 (39.6)
Hypertension	17 (60.7)	34 (57.6)	19 (54.3)	33 (68.8)
Diabetes	10 (35.7)	11 (18.6)	10 (28.6)	20 (41.7)
Overweight	10 (35.7)	16 (27.1)	10 (28.6)	13 (27.1)
Familiarity	11 (39.3)	18 (30.5)	13 (37.1)	16 (33.3)
**Cardiological clinical history and/or index event**				
Recent or previous AMI	12 (42.9)	26 (44.1)	18 (51.4)	14 (29.2)
Angioplasty	13 (46.4)	19 (32.2)	7 (20.0)	5 (10.4)
Revascularization	1 (3.6)	15 (25.4)	–	1 (2.1)
Valvulopathy (valve replacement)	1 (3.6)	12 (20.3)	2 (5.7)	5 (10.4)
Chronic obstructive arteriopathy	–	2 (3.4)	1 (2.9)	–
Heart transplantation	9 (32.1)	9 (15.3)	1 (2.9)	–
Chronic heart failure	8 (28.6)	14 (23.7)	2 (5.7)	6 (12.5)

*BMI, body mass index; AMI, acute myocardial infarction; Age^*a*^ < Age^*b*^, *p* < 0.001; Age^*c*^ < Age^*d*^, *p* < 0.0001.*

**TABLE 2:** Frequencies of selection criteria for in-depth psychological evaluation (PCS-Yes).

	2010 *n* (%)	2017 *n* (%)	χ^2^ *p*-Value
PCS-Yes	28 (32.2)	35 (42.2)	0.1778
**Selection criteria**			
Age (≤55 years)	10 (35.7)	11 (31.4)	0.7199
Anxiety	9 (32.1)	4 (11.4)	0.0435*
Depression	7 (25.0)	6 (17.1)	0.4438
Behavioral/psychiatric problems	2 (7.1)	–	0.1081
Cognitive disorders	1 (3.6)	5 (14.3)	0.0169*
Inadequate illness awareness	6 (21.4)	2 (5.7)	0.0627
Treatment non-adherence	8 (28.6)	7 (20.0)	0.4274
Current smoking	7 (25.0)	11 (31.4)	0.5746
Addiction	2 (7.1)	1 (2.9)	0.4274
Severe sleep disorders	–	–	–
Other (e.g., apathy, ambivalent behavior, extreme fear)	1 (3.6)	3 (8.7)	0.4187

*p < 0.05.

After PCS data comparison, several changes, integrations, and additions were made to the original schedule, to provide a revised version (Supplementary Data Sheets S1, S2) available in two languages. The PCS-R is divided into the following three sections:

– PCS-R (I)—Clinical and socio-demographic information, filled in by a trained nurse. A dedicated space is also provided for any notes or observations/clarifications concerning the patient’s clinical status (Pierobon et al., 2012).
– PCS-R (II)—Psychosocial checklist, filled in by a nurse previously trained by a psychologist. It has six sub-sections: psychological comorbidity manifestations, general/specific psychological and neuropsychological problems, disease management, social issues, caregiver needs, and positive affectivity. At the end of PCS-R (I-II), there is a sub-section where the interdisciplinary team (nurse, cardiologist, and psychologist) could indicate the need for further psychological/neuropsychological interview and/or testing (Pierobon et al., 2012; Sommaruga et al., 2018).
– PCS-R (III)—Psychological intervention, filled in by the psychologist after the psychological interview. It indicates the recognition of specific risk and protective psychosocial factors in the sensory, emotional, behavioral, cognitive, as well as interpersonal, social, and family areas. This section also specifies the technique(s) adopted during the psychological intervention (Bettinardi et al., 2014).

## Discussion

This retrospective comparison of PCS data collected in 2010 with data collected in 2017 provided an interesting overview of the evolution in the profile of cardiac patients attending CR, particularly concerning differences in socio-demographic characteristics, clinical variables, and the specific motivations for psychological counseling. In addition, it led to PCS-R adding more specific classifications regarding heart diseases and broadening the psychosocial investigation and intervention in a rehabilitative setting.

The results of the retrospective analysis of the PCS data highlighted the progressive aging of the cardiac patient population participating in CR, although the framework of social and cardiac clinical history characteristics remained quite similar. In fact, we found a significant difference in the mean age of the two samples and this is in line with the well-known aging population phenomenon (United Nations, 2017). Moreover, we found an increase in the number of male patients in 2017. This finding, however, does not fully reflect the latest epidemiological trends, which show that the prevalence of males in CV disease is declining while CV problems are increasing in females, above all in the older population (Pectoris, 2013; Townsend et al., 2016).

As concerns the patients’ current and past clinical cardiac characteristics and risk factors, there was no significant difference between the two samples. We found a modest increment (non-significant) in the number of PCS-Yes in 2017 with respect to 2010 (32.2 vs. 42.2%). This data might outline an increased attention of healthcare professionals on the issues presented in the latest cardiac (Forman and Wenger, 2013; Piepoli et al., 2016; Jackson et al., 2017) and psychosocial guidelines and position papers (Pogosova et al., 2015; Sommaruga et al., 2018; Albus et al., 2019b).

Concerning the criteria for an in-depth psychological interview, two significant differences were found. First, a significant decrease in reported signs of anxiety was unveiled. The detection of these anxiety signs may disappear behind the stronger influence of depression on self-care (Muller-Tasch et al., 2018) or other behavioral manifestations such as apathy, ambivalent behavior, or extreme fear that represented 8.7% of the selection criteria for in-depth psychological evaluation in 2017. Second, there was a significant increase in reported cognitive disorders. This is in line with the growing number of elderly patients (United Nations, 2017) who may require a specific neuropsychological assessment. These data could also be linked to the well-known associations between cardiac disease and cognitive issues (Pressler et al., 2010; Cameron et al., 2017). Even though the PCS is based simply upon observations by trained nurses, the data collected on the incidence of psychological manifestations (anxiety, depression), behavioral issues (smoking and addiction), and disease management (disease awareness and adherence to treatment) are in line with the most recent research and position papers (Pogosova et al., 2015; Sommaruga et al., 2018; Albus et al., 2019b).

Furthermore, in accordance with the recent CR guidelines and position papers, these results led to the revision of the schedule. As to PCS-R (I), data from literature offered the opportunity to provide a more specific and updated classification of heart diseases. As to PCS-R (II), the growing number of older adults and the reported necessity of a cognitive evaluation are often associated with an increasing need to provide a dedicated socio-familiar care pathway (Carmeli, 2014; Jaul and Barron, 2017; Grant and Graven, 2018). To this regard, the present data strengthened the paramount importance of evaluating the caregivers’ needs that were not specifically addressed in the original version of the PCS. Moreover, the reported patients’ psychological problems led to a rearrangement of the psychosocial checklist by adding more specific and updated issues (Supplementary Data Sheets S1, S2).

A psychological assessment/intervention creates a specific setting in which patients could report some additional and useful information that might be fundamental to enhance their treatment adherence and could help the interdisciplinary team to better tailor their rehabilitative program (Pogosova et al., 2015; Pedersen et al., 2017; Pedersen and Doyle, 2019). The early recognition of patient’s psychosocial problems and the subsequent psychological intervention can reduce, in turn, the burden of care for both nurses and cardiologists (Feo et al., 2018). It is clear that nurses would require a training program in which the psychologist teaches them how to accurately identify and collect all the necessary information for the schedule compilation. This could be quite tough if nurses are not motivated or perceive the screening as simply a further, perhaps useless, addition to their workload (Turner et al., 2017). In this regard, trained nurses could do the first screening along with their other standard data collection. Therefore, it would not increase their amount of work. Instead, it would save time for psychologists in screening patients and allow them to dedicate their time to interventions on the selected patients. The resulting decrease in the extra working hours of psychologists might lower hospital administrative costs. At the same time, the early recognition of not clinically relevant psychosocial problems could reduce the patient-related costs, by avoiding unnecessary time and money spent on interventions (Shields et al., 2018). Hence, the PCS-R might constitute an important asset for reducing healthcare costs.

Moreover, the PCS-R (III) highlights the patients’ challenging and protective factors, according to the biopsychosocial model applied in rehabilitation (Engel, 1977, 1980). Specifically, it suggests useful information about the psycho-educational intervention implemented, the strategies for preserving and/or stimulating cognitive capacities, and the eventual referral to psychiatric counseling and/or social services (Fattirolli et al., 2018; Sommaruga et al., 2018).

The PCS-R format is designed for easy digitalization. Hence, an accurate PCS-R data collection and subsequent digitalization, besides enhancing clinical practice, would be of use both in writing the psychological report and in providing information for clinical research (Giardini et al., 2018). The report could refer also to the patient and caregiver’s psychosocial evaluation and it could suggest relevant indications to improve disease awareness, management skills, and treatment adherence. Therefore, the PCS-R could be a handy tool to strengthen the communication and cooperation between the different professional figures of the interdisciplinary rehabilitation team. Moreover, it could allow an early classification of patients and caregivers’ needs and issues that is crucial in patient-centered interventions in both acute and chronic diseases (Korner et al., 2016; Fattirolli et al., 2018; Poitras et al., 2018; La Rosa et al., 2020).

This study has to be read in the light of its strengths and limitations. Concerning the PCS-R, several limitations could be highlighted: its complexity, the need for a real sharing of rehabilitative outcomes among the team, the necessity of specific training to detect psychosocial issues, and the professional’s high motivation to fill in the schedule. Another important limitation is the lack of updated PCS-R data, in particular those regarding the new sections on psychosocial needs, challenges, and psychotherapeutic intervention. Nevertheless, some points of strength are present too. First, the PCS-R allows the early recognition of patients’ psychosocial problems that may suggest tailored subsequent psychological interventions. Second, the report of the different psychological techniques adopted could also consent to better focus on patients’ needs, helping to reduce the burden of care for the interdisciplinary team. Finally, since the PCS-R is a screening checklist and not a diagnostic test, a validation process is not mandatory. Nevertheless, future studies, which are already in progress, could be useful to evaluate its feasibility and reliability in the hospital ward’s routine.

Conclusively, this study presents interesting screening data on the evolution of CR patients over time. Moreover, these data, in accordance with recent literature, led to a revision process of a well-established screening tool finalized to collect data that could be representative of the actual population attending CR. Indeed, the PCS-R might represent an ideal screening tool for the interdisciplinary team’s detection of psychosocial problems in patients undergoing CR that could help to tailor the psychological intervention according to the patient’s emotional, cognitive, and social needs.

## Data Availability Statement

The datasets generated for this study are available on request to the corresponding author.

## Ethics Statement

The studies involving human participants were reviewed and approved by the Review Board and Central Ethics Committee of the ICS Maugeri SpA SB (CEC). The patients/participants provided their written informed consent to participate in this study.

## Author Contributions

AP, VT, and MF did the data collection of the first PCS version. NG, EN, and MV managed the database and did the statistical analysis. AP, VT, MF, MS, EA, CR, and AC contributed to the conception of the new Italian and English version of the schedule (PCS-R). NG and AP wrote the manuscript. All authors contributed to the manuscript review and read and approved the submitted version.

## Conflict of Interest

The authors declare that the research was conducted in the absence of any commercial or financial relationships that could be construed as a potential conflict of interest.
