# Supplementary material for: Psychosocial Cardiological Schedule-Revised (PCS-R) in a Cardiac Rehabilitation Unit: Reflections Upon Data Collection (2010–2017) and New Challenges
Source: Front Psychol. 2020 Jul 14;11:1720. doi: 10.3389/fpsyg.2020.01720 (PMC7381208; doi:10.3389/fpsyg.2020.01720)
Supplement: Supplementary file 1 [file Data_Sheet_1.PDF]

PSYCHOSOCIAL CARDIOLOGICAL SCHEDULE – REVISED (PCS-R)

Cognome …………………………………………………………………**Nome**……………………………………**Sesso** ………**Età** (≤55) ………

Data di compilazione…………………………………………………**Data di ricovero**…………………………………………………………………….

Compilatore: operatore sanitario …………………………**Medico di riferimento** ……………………………………………………

**PCS-R (I) DATI CLINICI E SOCIO-ANAGRAFICI (a cura del professionista sanitario in reparto)**<br>(Pierobon et al., 2012, pag.95-96, modificata)	
**1. Stato civile**:<br>1. Libero<br>2. Coniugato-convivente<br>3. Vedovo<br>4. Separato<br>5. Divorziato<br><br>**2. Titolo di studio**:<br>1. Nessuno<br>2. Licenza elementare<br>3. Licenza scuola media inferiore<br>4. Diploma scuola media superiore<br>5. Diploma universitario<br>6. Laurea	**3. Attività lavorativa**:<br>1. Artigiano/commerciante<br>2. Casalinga<br>3. Disoccupato<br>4. Dirigente<br>5. Impiegato<br>6. Imprenditore<br>7. Insegnante<br>8. Libero professionista<br>9. Operaio<br>10. Invalido<br>11. Pensionato<br>12. Altro
**4. Con chi vive**: Barrare anche più di una risposta<br>1. Solo<br>2. Con coniuge-convivente<br>3. Con figli<br>4. Con coniuge e figli<br>5. Con parenti (non coniuge o figli)<br>6. Con altre persone (no familiari)	**5. Persona di riferimento per la cura**:<br>1. Coniuge<br>2. Figlio-figlia<br>3. Genitori<br>4. Altro familiare<br>5. Altra persona non di famiglia<br>6. Nessuno
**6. Ha avuto precedenti ricoveri in Cardiologia Riabilitativa?**<br>1. No 2. Si	
**7. Tipo di cardiopatia:** Barrare anche più di una risposta<br>1. Insufficienza cardiaca cronica<br>2. Portatore di CRT-D (terapia di resincronizzazione cardiaca con defibrillatore impiantabile)/ ICD<br>3. LVAD (Assistenza Ventricolare Sinistra)<br>4. Trapianto di cuore (anno…………..)<br>5. Sindrome coronarica acuta<br>6. Sindrome coronarica cronica<br>7. Angioplastica coronarica con/senza stent<br>8. Bypass aorto-coronarico<br>9. Sostituzione o riparazione valvolare<br>10.Malattia arteriosa periferica<br>11.Altro (……………………………………………)	**8. Anamnesi fattori di rischio:**<br>Barrare anche più di una risposta<br>1. Fumo<br>2. Dislipidemia<br>3. Ipertensione arteriosa<br>4. Diabete<br>5. Iperuricemia<br>6. Sovrappeso<br>7. Potus<br>8. Dipendenza da sostanze<br>9. Familiarità
**9. Comorbilità:**<br>……	
**10. Durata della malattia cardiaca**: N…………….<br>(Anni, mesi o gg dall'esordio)	**11. BMI**<br><br>**peso**: (Kg)…………<br><br>**altezza**: (cm)……….
**Ulteriori dati clinici:**<br>……<br>……	

(A cura di) Pierobon A[a], Ferrari M[a], Giardini A[a], Torlaschi V[a], Sommaruga M [a], Angelino E[a], Rizza C[b]
[a]Istituti Clinici Scientifici Maugeri, IRCCS, Pavia; [b]Gruppo Multimedica Spa-IRCCS, Milano.

**PCS-R (II) CHECKLIST PSICO-SOCIALE (a cura del professionista sanitario formato)**<br>(Pierobon et al., 2012, pag.95-96, modificata; Sommaruga et al., 2018, pag.72, modificata)	
**13. Manifestazioni psicologiche in comorbidità**<br>☐ Depressione[1]<br>☐ Ansia[2]<br>☐ Disturbo da stress post-traumatico[3]	■
**14. Problemi psicologici generale/specifici**<br>☐ Aspetti personologici: □ Personalità di tipo D □ Ostilità[4]<br>☐ Disturbo di dipendenza attuale o passato: □ tabacco □ alcool □ altre sostanze (…………)<br>☐ Disturbo neuropsicologico[5]<br>☐ Disturbo da stress post-traumatico cronico (il paziente riferisce eventi negativi o traumatici)<br>☐ Stress cronico lavoro correlato[6]<br>☐ Problemi riferiti in ambito sessuale (qualche tipo di disfunzione sessuale?)<br>☐ Gravi disturbi del sonno<br>☐ Disturbi psichiatrici in anamnesi e/o trattamenti presenti presso i servizi psichiatrici locali di competenza[7]<br>Terapia psicofarmacologica in atto………………………………………………………………………………………………..	■
**15. Gestione della malattia**<br>☐ Inadeguata consapevolezza e accettazione della malattia<br>☐ Il paziente mostra segni di intolleranza/minimizzazione/non aderenza riguardo alle prescrizioni cliniche:<br>☐ farmacologiche<br>☐ tabagiche e/o altre dipendenze<br>☐ dietetiche/alimentari<br>☐ fisioterapiche	■
**16. Difficoltà socio-familiari**<br>☐ Isolamento sociale<br>☐ Basso livello socio-economico[8]<br>☐ Carente supporto socio-familiare	■
**17. Bisogni di Caregiver**<br>☐ Bisogni del caregiver<br>☐ Caregiver burden[9]	■
**18. Affettività positiva**<br>☐ Sentirsi fiduciosi e competenti nei confronti della propria condizione di salute[10]<br>☐ Sereno e ottimista[11]	☐

Le caselle selezionate e in grigio indicano la necessità di una valutazione psicosociale clinica approfondita (a cura del team interdisciplinare)

Suggerimenti per un'ulteriore valutazione psicologica ospedaliera o invio ai servizi sociali/psichiatrici territoriali

☐ Si - Approfondimento clinico
☐ Si - Approfondimento clinico e testistico (psicologico/neuropsicologico)
☐ Si - Invio gruppi psico-educazionali
☐ No

[1] Durante l'ultimo mese il paziente si è sentito giù e/o senza speranza?

[2] Durante l'ultimo mese il paziente ha avuto paure improvvise o panico? Spesso non è in grado di fermare o controllare le preoccupazioni?

[3] Durante l'ultimo mese il paziente è stato esposto a un evento traumatico? Soffre di incubi o pensieri intrusivi?

[4] In generale, il paziente tende ad evitare di condividere i suoi pensieri e sentimenti con altre persone?

[5] Il paziente mostra segni di compromissione cognitiva? Presenta segni di disorientamento spazio-temporale, personale e familiare?

[6] Il paziente fa riferimento a lunghi orari di lavoro, lavoro straordinario, elevate esigenze psicologiche, ingiustizia e tensione lavorativa?

[7] Il paziente ha sintomi psicologici remoti che influenzano l'effettiva gestione o accettazione delle malattie cardiache?

[8] Il paziente vive da solo e in una zona residenziale povera? Ha un basso livello di istruzione e un basso reddito?

[9] Il caregiver ha bisogno di informazioni e supporto emotivo? La cura dei pazienti è percepita come esigente e caratterizzata da un forte disagio emotivo e da problemi economici?

[10] Durante l'ultimo mese il paziente si è sentito fiducioso? Si sente in grado di gestire i cambiamenti delle condizioni di salute?

[11] Durante l'ultimo mese il paziente si è sentito ottimista e sereno nell'affrontare le sue condizioni di salute?

(A cura di) Pierobon A[a], Ferrari M[a], Giardini A[a], Torlaschi V[a], Sommaruga M [a], Angelino E[a], Rizza C[b]
[a]Istituti Clinici Scientifici Maugeri, IRCCS, Pavia; [b]Gruppo Multimedica Spa-IRCCS, Milano.

Cognome ……………………………………………………………………**Nome**………………………………………………………………………..

PCS-R (III) INTERVENTO PSICOLOGICO (a cura dello psicologo)
Aree psicosociali problematici e/o fattori protettivi rilevati durante l'intervento psicologico
(Bettinardi et al., 2014, pag.144, modificata)

**AREE**	**PROBLEMI PSICOSOCIALI**		**FATTORI PROTTETIVI**	
**Sensoriali**	Tensione muscolare, dolore acuto e cronico, fragilità, debolezza, affaticamento, difficoltà respiratorie, problemi di sonno	☐	Consapevolezza corpo-mente, energia, forza, creatività e capacità artistiche	☐
**Emotive**	Depressione, ansia, angoscia, alessitimia, fattori traumatici	☐	Affettività positiva, riconoscimento e gestione delle reazioni emotive, stabilità emotiva, strategie attive focalizzate sul problema	☐
**Comportamentali**	Utilizzo di sostanze, disturbi alimentari, sedentarietà, scarsa aderenza alle prescrizioni cliniche	☐	Stile di vita finalizzato al mantenimento della salute, aderenza ai trattamenti farmacologici, competenze assertive, comportamenti volti al "problem solving"	☐
**Cognitive**	Scarsa conoscenza dei fattori di rischio della malattia, difficoltà di elaborazione e di percezione della malattia, difficoltà ad accettare la malattia, preoccupazione, decadimento cognitivo	☐	Risorse cognitive e culturali, resilienza, conoscenza della malattia e fattori di rischio associati, controllo interno, problem-solving, autoefficacia, motivazione interna	☐
**Interpersonali/ sociali/famiglia**	Problemi relazionali, problemi riguardanti le attività lavorative, problemi sociali, problemi sessuali, supporto socio-familiare scarso o insoddisfacente	☐	Rete di supporto familiare, rete di supporto sociale, attività lavorativa adeguata, contesto socio-economico adeguato	☐

**TECNICHE PSICOLOGICHE**		
**Supportive**	Rafforzamento delle risorse, interne ed esterne, e elaborazione delle emozioni dolorose	☐
**Comportamentali**	Bilanciamento dei comportamenti funzionali e disfunzionali	☐
**Cognitive**	Ristrutturazione cognitiva	☐
**Gestione dello stress**	Autocontrollo, cura di sé, capacità comunicative e rilassamento	☐
**Tecniche mind-body**	Aumento della consapevolezza/accettazione e auto-regolazione del corpo	☐

Note psicologiche

………

………

………

………

………

………
