# Supplementary material for: Psychosocial Cardiological Schedule-Revised (PCS-R) in a Cardiac Rehabilitation Unit: Reflections Upon Data Collection (2010–2017) and New Challenges
Source: Front Psychol. 2020 Jul 14;11:1720. doi: 10.3389/fpsyg.2020.01720 (PMC7381208; doi:10.3389/fpsyg.2020.01720)
Supplement: Supplementary file 2 [file Data_Sheet_2.PDF]

PSYCHOSOCIAL CARDIOLOGICAL SCHEDULE - REVISED (PCS-R)

Surname ....**Name**..**Sex** .... **Age** (≤55)..............

Date of admission...................................... **Compilation date**...

Healthcare operator...................................... **Referring physician**..

PCS-R (I) CLINICAL AND SOCIO-DEMOGRAPHIC INFORMATION (filled in by healthcare professional)
(Pierobon et al., 2012, pag.95-96, modified)

1. Family status:
1. Unmarried
2. Married/Partner
3. Widow/widower
4. Separated
5. Divorced

2. Education:
1. None
2. Less than 5 years
3. 6 – 8 years of schooling
4. 9 – 13 years of schooling
5. More than 14 years or University Degree

3. Current occupation:
1. Craftsman/Shopkeeper
2. Staying with parent/Houseman or Housewife
3. Unemployed
4. Manager
5. Employee
6. Businessman
7. Teacher
8. Self-employed
9. Worker
10. Disabled
11. Retired
12. Other

4. Whom do you live with:
Check more than one box, if necessary
1. By myself
2. Husband/wife/partner
3. Children
4. Husband/wife/partner and children
5. Other family members (apart from 4)
6. Other people (non-family)

5. Primary caregiver:
1. Husband/wife/partner
2. Son/Daughter
3. Parents
4. Other family member
5. Other person (not family member)
6. Nobody

6. Have you been previously admitted to Cardiac Rehabilitation?
1. No 2. Yes

7. Type of heart disease:
Check more than one box, if necessary
1. Chronic Heart Failure
2. CRT-D (implantable cardiac resynchronization therapy defibrillator)/ICD
3. LVAD (left ventricular assist device)
4. Heart transplantation (year...........)
5. ACS (Acute Coronary Syndrome)
6. CCS (Chronic Coronary Syndrome)
7. PTCA (percutaneous transluminal coronary angioplasty with or without stent)
8. CABG (coronary Artery bypass grafting)
9. Valvulopathy (Valve replacement/surgery)
10. PAD (peripheral artery disease)
11. Other (...)

8. Anamnesis risk factors
Please check more than one box, if necessary
1. Smoking
2. Dyslipidemia
3. Hypertension
4. Diabetes
5. Hyperuricemia
6. Overweight
7. Alcohol abuse
8. Drug addiction
9. Cardiovascular family history

9. Comorbidity:
....

10. Duration of cardiac illness: N................
(years, months or days)

11. BMI...............

Weight: (Kg)............

Height: (cm)..........

Any extra clinical notes
....
....

(Edited by) Pierobon A[a], Ferrari M[a], Giardini A[a], Torlaschi V[a], Sommaruga M [a], Angelino E[a], Rizza C[b]
[a]Istituti Clinici Scientifici Maugeri, IRCCS, Pavia; [b]Gruppo Multimedica Spa-IRCCS, Milano, Italy.

**PCS-R (II) PSYCHOSOCIAL CHECKLIST (filled in by a trained healthcare professional)**<br>(Pierobon et al., 2012, pag.95-96, modified; Sommaruga et al., 2018, pag.72, modified)	
**13. Psychological comorbidities manifestations**<br>☐ Depression[1]<br>☐ Anxiety[2]<br>☐ Post-traumatic stress disorder[3]	■
**14. General/specific psychological problems**<br>☐ Personality traits: □ Type D Personality □ Hostility[4]<br>☐ Actual or past dependence disorder: □ tobacco □ alcohol □ other substances (………)<br>☐ Neuropsychological disorder[5]<br>☐ Chronic post-traumatic stress (if the patient reports negative or traumatic life events)<br>☐ Chronic work stress[6]<br>☐ Problems regarding the area of sexuality (any type of sexual dysfunction?)<br>☐ Severe sleep disorders<br>☐ Past psychiatric disorder and/or under current treatment at local psychiatric services[7]<br>Actual psychopharmacological treatment …………………………………………………………………………………………………….	■
**15. Disease management**<br>☐ Inadequate awareness and acceptance of the disease<br>☐ The patient shows signs of intolerance/minimization/non-adherence regarding clinical prescriptions:<br>☐ medications<br>☐ smoking and/or other addictions<br>☐ dietary<br>☐ physical activity	■
**16. Social issues**<br>☐ Social isolation<br>☐ Low socio-economic status[8]<br>☐ Lack of socio-familiar support	■
**17. Caregiver needs**<br>☐ Caregiver needs<br>☐ Caregiver burden[9]	■
**18. Positive Affectivity**<br>☐ Trustful and competent abilities regarding health conditions[10]<br>☐ Serene and optimistic[11]	☐

The answers with checked boxes indicate the need for a clinical psychosocial in-depth evaluation (filled in by the interdisciplinary team)

Suggestions for further inpatient psychological evaluation or referral to local social/psychiatric support services

- ☐ Yes – Clinical interview
- ☐ Yes – Clinical interview and psychometric (psychological/neuropsychological) evaluation
- ☐ Yes – Psycho-educational groups
- ☐ No

---

[1] During the last month, did the patient feel down and/or hopeless? Did he/she lose interest and pleasure in life?
[2] During the last month, did the patient experience sudden fears or panic? Is he/she frequently unable to stop or control worrying?
[3] During the last month, has the patient experienced a traumatic event? Does he/she suffer from nightmares or intrusive thoughts?
[4] In general, does he/she tend to avoid sharing his thoughts and negative feelings with other people?
[5] Does the patient show signs of cognitive impairment? Does he/she show signs of personal, family, spatial/temporal disorientation?
[6] Does the patient report long working hours, extensive overtime work, high psychological demands, unfairness at work and job strain?
[7] Does the patient have remote psychological symptoms that influence the cardiac disease management or acceptance?
[8] Does the patient live alone, in a poor residential area? Has he/she a low educational level, a low income?
[9] Does the caregiver need information, emotional support? Is the care of the patient perceived as demanding and involve strong emotional distress and economic problems?
[10] During the last month, did the patient feel hopeful? Does the patient feel able to manage the changes in his/her health conditions?
[11] During the last month, the patient felt optimistic and serene in dealing with his/her health conditions?

Surname ……………………………………………………………………………**Name**…………………………………………………………….

**PCS-R (III) PSYCHOLOGICAL INTERVENTION (filled in by the Psychologist)**<br>**Psychosocial challenging areas and/or protective factors detected during the psychological intervention**<br>(Bettinardi et al., 2014, pag.144, modified)				
**AREAS**	**PSYCHOSOCIAL CHALLENGES**		**PROTECTIVE FACTORS**	
**Sensory**	Muscle tension, chronic and acute pain, frailty, weakness, fatigue, breathing difficulty, sleep problems	☐	Mind-body awareness, energy, strength, creativity and arts	☐
**Emotional**	Depression, anxiety, distress, alexithymia aspects, traumatic factors	☐	Positive affectivity, recognition and management of emotional reactions, emotional stability, active strategies aimed at focusing on problem	☐
**Behavioral**	Substance abuse, eating disorders, sedentary lifestyle, low adherence to clinical prescriptions	☐	Lifestyle aimed at maintaining health, adherence to pharmacological treatments, assertive skills, behaviors aimed at problem-solving	☐
**Cognitive**	Poor knowledge of the disease risk factors, difficulty in processing/ perception of the disease, difficulty accepting the disease, worry, cognitive decline	☐	Cognitive and cultural resources, resilience, knowledge of illness and associated risk factors, internal locus of control, problem-solving, self-efficacy, mastery motivation	☐
**Interpersonal/ social/family**	Relational problems, problems concerning work activities, social problems, sexual problems, poor or unsatisfactory socio-family support	☐	Family reference support-network, social reference support-network, adequate work activity, adequate socio-economic context)	☐
**PSYCHOLOGICAL TECHNIQUES**				
**Supportive**	Strengthening of external and internal resources and processing of painful emotions			☐
**Behavioral**	Balancing of dysfunctional and functional behaviors			☐
**Cognitive**	Cognitive restructuring			☐
**Stress management**	Self-control, self-care, communication skills and relaxation			☐
**Mind-body techniques**	Increasing awareness/acceptance and body regulation			☐

Psychological notes

……

……

……

……

……

(Edited by) Pierobon A[a], Ferrari M[a], Giardini A[a], Torlaschi V[a], Sommaruga M [a], Angelino E[a], Rizza C[b]
[a]Istituti Clinici Scientifici Maugeri, IRCCS, Pavia; [b]Gruppo Multimedica Spa-IRCCS, Milano, Italy.
